# Supplementary material for: Efficient SARS-CoV-2 Surveillance during the Pandemic-Endemic Transition Using PCR-Based Genotyping Assays
Source: Microbiol Spectr. 2023 May 8;11(3):e03450-22. doi: 10.1128/spectrum.03450-22 (PMC10269661; doi:10.1128/spectrum.03450-22)
Supplement: Supplemental file 1 — Tables S1 to S3. Download spectrum.03450-22-s0001.pdf, PDF file, 0.3 MB [file spectrum.03450-22-s0001.pdf]

Supplementary Table S1A: Accession numbers of the SARS-CoV-2 sequences with 15≤ C_T ≤32 (N=664).

Sample number	GISAID Accession ID
1	EPI_ISL_3486632
2	EPI_ISL_3486634
3	EPI_ISL_3650583
4	EPI_ISL_3650589
5	EPI_ISL_3650596
6	EPI_ISL_3650590
7	EPI_ISL_3650584
8	EPI_ISL_3650585
9	EPI_ISL_3650586
10	EPI_ISL_3650581
11	EPI_ISL_3650588
12	EPI_ISL_3650591
13	EPI_ISL_3650592
14	EPI_ISL_3838697
15	EPI_ISL_3650597
16	EPI_ISL_3650593
17	EPI_ISL_3650594
18	EPI_ISL_3650598
19	EPI_ISL_3650599
20	EPI_ISL_3650600
21	EPI_ISL_3650595
22	EPI_ISL_3650601
23	EPI_ISL_3650602
24	EPI_ISL_3650603
25	EPI_ISL_3650552
26	EPI_ISL_3650553
27	EPI_ISL_3650554
28	EPI_ISL_3650555
29	EPI_ISL_3650556
30	EPI_ISL_3650557
31	EPI_ISL_3650605
32	EPI_ISL_3650568
33	EPI_ISL_3650569
34	EPI_ISL_3650570
35	EPI_ISL_3650571
36	EPI_ISL_3650604
37	EPI_ISL_3650572
38	EPI_ISL_3650558
39	EPI_ISL_3650573
40	EPI_ISL_3650559
41	EPI_ISL_3650574
42	EPI_ISL_3650575
43	EPI_ISL_3650606
44	EPI_ISL_3650607
45	EPI_ISL_3650608
46	EPI_ISL_3650609
47	EPI_ISL_3650610
48	EPI_ISL_3650611
49	EPI_ISL_3650560
50	EPI_ISL_3650561
51	EPI_ISL_3650562
52	EPI_ISL_3650563
53	EPI_ISL_3654197
54	EPI_ISL_3654198
55	EPI_ISL_3650576
56	EPI_ISL_3654199
57	EPI_ISL_3654190
58	EPI_ISL_3654200
59	EPI_ISL_3654191
60	EPI_ISL_3654192
61	EPI_ISL_3654201
62	EPI_ISL_3838703
63	EPI_ISL_3650577
64	EPI_ISL_3650578
65	EPI_ISL_3838704
66	EPI_ISL_3838701
67	EPI_ISL_3650564
68	EPI_ISL_3650579
69	EPI_ISL_3654193
70	EPI_ISL_3654194
71	EPI_ISL_3650565
72	EPI_ISL_3650580
73	EPI_ISL_3650498
74	EPI_ISL_3654195
75	EPI_ISL_3654202
76	EPI_ISL_3650566
77	EPI_ISL_3650581
78	EPI_ISL_3654196
79	EPI_ISL_3650582
80	EPI_ISL_3650499
81	EPI_ISL_3650486
82	EPI_ISL_3838702
83	EPI_ISL_3650487
84	EPI_ISL_3650489
85	EPI_ISL_3650490
86	EPI_ISL_3650567
87	EPI_ISL_3650491
88	EPI_ISL_3654203
89	EPI_ISL_3650500
90	EPI_ISL_3650492
91	EPI_ISL_3838700
92	EPI_ISL_3650493
93	EPI_ISL_3650494
94	EPI_ISL_3650495
95	EPI_ISL_3650496
96	EPI_ISL_3650497
97	EPI_ISL_3650501
98	EPI_ISL_3650488
99	EPI_ISL_3650480
100	EPI_ISL_3650502
101	EPI_ISL_3650481
102	EPI_ISL_3838698
103	EPI_ISL_3650482
104	EPI_ISL_3650483
105	EPI_ISL_3838699
106	EPI_ISL_3650484
107	EPI_ISL_3650485
108	EPI_ISL_3654204
109	EPI_ISL_3823154
110	EPI_ISL_3654205
111	EPI_ISL_3654206
112	EPI_ISL_3654207
113	EPI_ISL_3654219
114	EPI_ISL_3654208
115	EPI_ISL_3654220
116	EPI_ISL_3654211
117	EPI_ISL_3654212
118	EPI_ISL_3654209
119	EPI_ISL_3654221
120	EPI_ISL_3654213
121	EPI_ISL_3654214
122	EPI_ISL_3654222
123	EPI_ISL_3654223
124	EPI_ISL_3654227
125	EPI_ISL_3654228
126	EPI_ISL_3654229
127	EPI_ISL_3654224

128	EPI_ISL_3654215
129	EPI_ISL_3654225
130	EPI_ISL_3654226
131	EPI_ISL_3654230
132	EPI_ISL_3654235
133	EPI_ISL_3654216
134	EPI_ISL_3654210
135	EPI_ISL_3654231
136	EPI_ISL_3654236
137	EPI_ISL_3654237
138	EPI_ISL_3654217
139	EPI_ISL_3654243
140	EPI_ISL_3654238
141	EPI_ISL_3654232
142	EPI_ISL_3654233
143	EPI_ISL_3654244
144	EPI_ISL_3654245
145	EPI_ISL_3654246
146	EPI_ISL_3654239
147	EPI_ISL_3654240
148	EPI_ISL_3654247
149	EPI_ISL_3654241
150	EPI_ISL_3654248
151	EPI_ISL_3654249
152	EPI_ISL_3654242
153	EPI_ISL_3654234
154	EPI_ISL_3654250
155	EPI_ISL_3654218
156	EPI_ISL_3654273
157	EPI_ISL_3654251
158	EPI_ISL_3654252
159	EPI_ISL_3654253
160	EPI_ISL_3654254
161	EPI_ISL_3654255
162	EPI_ISL_3654266
163	EPI_ISL_3654259
164	EPI_ISL_3654267
165	EPI_ISL_3654256
166	EPI_ISL_3654268
167	EPI_ISL_3654257
168	EPI_ISL_3838198
169	EPI_ISL_3654260
170	EPI_ISL_3654261
171	EPI_ISL_3654269
172	EPI_ISL_3654274

173	EPI_ISL_3654270
174	EPI_ISL_3654258
175	EPI_ISL_3654262
176	EPI_ISL_3654271
177	EPI_ISL_3654263
178	EPI_ISL_3654264
179	EPI_ISL_3654265
180	EPI_ISL_3654272
181	EPI_ISL_3838199
182	EPI_ISL_3654275
183	EPI_ISL_3654276
184	EPI_ISL_3654277
185	EPI_ISL_3654278
186	EPI_ISL_3654279
187	EPI_ISL_3654280
188	EPI_ISL_3650851
189	EPI_ISL_3838173
190	EPI_ISL_3650859
191	EPI_ISL_3650852
192	EPI_ISL_3650860
193	EPI_ISL_3650861
194	EPI_ISL_3650866
195	EPI_ISL_3650867
196	EPI_ISL_3850853
197	EPI_ISL_3650862
198	EPI_ISL_3650854
199	EPI_ISL_3650863
200	EPI_ISL_3650855
201	EPI_ISL_3650856
202	EPI_ISL_3650857
203	EPI_ISL_3650868
204	EPI_ISL_3650864
205	EPI_ISL_3650858
206	EPI_ISL_3650869
207	EPI_ISL_3650865
208	EPI_ISL_3650873
209	EPI_ISL_3650874
210	EPI_ISL_3650875
211	EPI_ISL_3838174
212	EPI_ISL_3650870
213	EPI_ISL_3650876
214	EPI_ISL_3650877
215	EPI_ISL_3650871
216	EPI_ISL_3650878
217	EPI_ISL_3650879

218	EPI_ISL_3650872
219	EPI_ISL_3650880
220	EPI_ISL_3650681
221	EPI_ISL_3650670
222	EPI_ISL_3650646
223	EPI_ISL_3650682
224	EPI_ISL_3650683
225	EPI_ISL_3650671
226	EPI_ISL_3650647
227	EPI_ISL_3650684
228	EPI_ISL_3650659
229	EPI_ISL_3650660
230	EPI_ISL_3650661
231	EPI_ISL_3650662
232	EPI_ISL_3650648
233	EPI_ISL_3650544
234	EPI_ISL_3650536
235	EPI_ISL_3650537
236	EPI_ISL_3650519
237	EPI_ISL_3650545
238	EPI_ISL_3650538
239	EPI_ISL_3650672
240	EPI_ISL_3650546
241	EPI_ISL_3650649
242	EPI_ISL_3650520
243	EPI_ISL_3650547
244	EPI_ISL_3650548
245	EPI_ISL_3650539
246	EPI_ISL_3650549
247	EPI_ISL_3650550
248	EPI_ISL_3650521
249	EPI_ISL_3650522
250	EPI_ISL_3650523
251	EPI_ISL_3650540
252	EPI_ISL_3650541
253	EPI_ISL_3650551
254	EPI_ISL_3650524
255	EPI_ISL_3650650
256	EPI_ISL_3650542
257	EPI_ISL_3650543
258	EPI_ISL_3650527
259	EPI_ISL_3650528
260	EPI_ISL_3650529
261	EPI_ISL_3650530
262	EPI_ISL_3650531

263	EPI_ISL_3650532
264	EPI_ISL_3650533
265	EPI_ISL_3650525
266	EPI_ISL_3650526
267	EPI_ISL_3650534
268	EPI_ISL_3823275
269	EPI_ISL_3650663
270	EPI_ISL_3650654
271	EPI_ISL_3650655
272	EPI_ISL_3650641
273	EPI_ISL_3650656
274	EPI_ISL_3650685
275	EPI_ISL_3650686
276	EPI_ISL_3650651
277	EPI_ISL_3650657
278	EPI_ISL_3650796
279	EPI_ISL_3650780
280	EPI_ISL_3650673
281	EPI_ISL_3650781
282	EPI_ISL_3650769
283	EPI_ISL_3650642
284	EPI_ISL_3650652
285	EPI_ISL_3650674
286	EPI_ISL_3650678
287	EPI_ISL_3650782
288	EPI_ISL_3650667
289	EPI_ISL_3650770
290	EPI_ISL_3650653
291	EPI_ISL_3650668
292	EPI_ISL_3650783
293	EPI_ISL_3650675
294	EPI_ISL_3650679
295	EPI_ISL_3650676
296	EPI_ISL_3650643
297	EPI_ISL_3650677
298	EPI_ISL_3650784
299	EPI_ISL_3650644
300	EPI_ISL_3650645
301	EPI_ISL_3650771
302	EPI_ISL_3650669
303	EPI_ISL_3650664
304	EPI_ISL_3650680
305	EPI_ISL_3650687
306	EPI_ISL_3650665
307	EPI_ISL_3650666

308	EPI_ISL_3650658
309	EPI_ISL_3650777
310	EPI_ISL_3650797
311	EPI_ISL_3650778
312	EPI_ISL_3650779
313	EPI_ISL_3650798
314	EPI_ISL_3650799
315	EPI_ISL_3650800
316	EPI_ISL_3650772
317	EPI_ISL_3650773
318	EPI_ISL_3650785
319	EPI_ISL_3650786
320	EPI_ISL_3650774
321	EPI_ISL_3650789
322	EPI_ISL_3650787
323	EPI_ISL_3650790
324	EPI_ISL_3650775
325	EPI_ISL_3650791
326	EPI_ISL_3650788
327	EPI_ISL_3650792
328	EPI_ISL_3650793
329	EPI_ISL_3650794
330	EPI_ISL_3650795
331	EPI_ISL_3650776
332	EPI_ISL_7593002
333	EPI_ISL_7592988
334	EPI_ISL_7592987
335	EPI_ISL_7592967
336	EPI_ISL_7592997
337	EPI_ISL_7592978
338	EPI_ISL_7592968
339	EPI_ISL_7592990
340	EPI_ISL_7592984
341	EPI_ISL_7592956
342	EPI_ISL_7592960
343	EPI_ISL_7592975
344	EPI_ISL_7592983
345	EPI_ISL_7592980
346	EPI_ISL_7592969
347	EPI_ISL_7592957
348	EPI_ISL_7592998
349	EPI_ISL_7592993
350	EPI_ISL_7592977
351	EPI_ISL_7592982
352	EPI_ISL_7649110

353	EPI_ISL_7592974
354	EPI_ISL_7592995
355	EPI_ISL_7592996
356	EPI_ISL_7592981
357	EPI_ISL_7592951
358	EPI_ISL_7592955
359	EPI_ISL_7592994
360	EPI_ISL_7592966
361	EPI_ISL_7592965
362	EPI_ISL_7593003
363	EPI_ISL_7592976
364	EPI_ISL_7592952
365	EPI_ISL_7592953
366	EPI_ISL_7592947
367	EPI_ISL_7592985
368	EPI_ISL_7592986
369	EPI_ISL_7592979
370	EPI_ISL_7592992
371	EPI_ISL_7592950
372	EPI_ISL_7592991
373	EPI_ISL_7592959
374	EPI_ISL_7592989
375	EPI_ISL_7592949
376	EPI_ISL_7592958
377	EPI_ISL_7592954
378	EPI_ISL_7592948
379	EPI_ISL_7592999
380	EPI_ISL_7826340
381	EPI_ISL_7826358
382	EPI_ISL_7826378
383	EPI_ISL_7826348
384	EPI_ISL_7826335
385	EPI_ISL_7826372
386	EPI_ISL_7826350
387	EPI_ISL_7826364
388	EPI_ISL_7826357
389	EPI_ISL_7826360
390	EPI_ISL_7826352
391	EPI_ISL_7826341
392	EPI_ISL_7826356
393	EPI_ISL_7826365
394	EPI_ISL_7826368
395	EPI_ISL_7826337
396	EPI_ISL_7826338
397	EPI_ISL_7826344

398	EPI_ISL_7826377
399	EPI_ISL_7826342
400	EPI_ISL_7826345
401	EPI_ISL_7826354
402	EPI_ISL_7826343
403	EPI_ISL_7826367
404	EPI_ISL_7869638
405	EPI_ISL_7826371
406	EPI_ISL_7826373
407	EPI_ISL_7826351
408	EPI_ISL_7826366
409	EPI_ISL_7826363
410	EPI_ISL_7826362
411	EPI_ISL_7826361
412	EPI_ISL_7826359
413	EPI_ISL_7826370
414	EPI_ISL_7826346
415	EPI_ISL_7869640
416	EPI_ISL_7826353
417	EPI_ISL_7826375
418	EPI_ISL_7826339
419	EPI_ISL_7826369
420	EPI_ISL_7869639
421	EPI_ISL_7826347
422	EPI_ISL_7826349
423	EPI_ISL_7826374
424	EPI_ISL_7826355
425	EPI_ISL_7869641
426	EPI_ISL_7826336
427	EPI_ISL_7826376
428	EPI_ISL_8104076
429	EPI_ISL_8108892
430	EPI_ISL_8104105
431	EPI_ISL_8104073
432	EPI_ISL_8104090
433	EPI_ISL_8104065
434	EPI_ISL_8104078
435	EPI_ISL_8104101
436	EPI_ISL_8104071
437	EPI_ISL_8104089
438	EPI_ISL_8104072
439	EPI_ISL_8104108
440	EPI_ISL_8104099
441	EPI_ISL_8104106
442	EPI_ISL_8104102

443	EPI_ISL_8104074
444	EPI_ISL_8104087
445	EPI_ISL_8104070
446	EPI_ISL_8104075
447	EPI_ISL_8104081
448	EPI_ISL_8104104
449	EPI_ISL_8104096
450	EPI_ISL_8104092
451	EPI_ISL_8108893
452	EPI_ISL_8104110
453	EPI_ISL_8104085
454	EPI_ISL_8104100
455	EPI_ISL_8104109
456	EPI_ISL_8104066
457	EPI_ISL_8104103
458	EPI_ISL_8104079
459	EPI_ISL_8104077
460	EPI_ISL_8104097
461	EPI_ISL_8104098
462	EPI_ISL_8104069
463	EPI_ISL_8104094
464	EPI_ISL_8104083
465	EPI_ISL_8104093
466	EPI_ISL_8104095
467	EPI_ISL_8104082
468	EPI_ISL_8104068
469	EPI_ISL_8104084
470	EPI_ISL_8104091
471	EPI_ISL_8104088
472	EPI_ISL_8104080
473	EPI_ISL_8104067
474	EPI_ISL_8104086
475	EPI_ISL_8104107
476	EPI_ISL_8383183
477	EPI_ISL_8383206
478	EPI_ISL_8383200
479	EPI_ISL_8383207
480	EPI_ISL_8383202
481	EPI_ISL_8383205
482	EPI_ISL_8383211
483	EPI_ISL_8383177
484	EPI_ISL_8386761
485	EPI_ISL_8383187
486	EPI_ISL_8383217
487	EPI_ISL_8383214

488	EPI_ISL_8386762
489	EPI_ISL_8383209
490	EPI_ISL_8383203
491	EPI_ISL_8383208
492	EPI_ISL_8386765
493	EPI_ISL_8386760
494	EPI_ISL_8383180
495	EPI_ISL_8383186
496	EPI_ISL_8383179
497	EPI_ISL_8383188
498	EPI_ISL_8383182
499	EPI_ISL_8383191
500	EPI_ISL_8383178
501	EPI_ISL_8383185
502	EPI_ISL_8383210
503	EPI_ISL_8383204
504	EPI_ISL_8386764
505	EPI_ISL_8383213
506	EPI_ISL_8383193
507	EPI_ISL_8383189
508	EPI_ISL_8383192
509	EPI_ISL_8383199
510	EPI_ISL_8383195
511	EPI_ISL_8383196
512	EPI_ISL_8383198
513	EPI_ISL_8383190
514	EPI_ISL_8383194
515	EPI_ISL_8383181
516	EPI_ISL_8383184
517	EPI_ISL_8383197
518	EPI_ISL_8383212
519	EPI_ISL_8383215
520	EPI_ISL_8383216
521	EPI_ISL_8383201
522	EPI_ISL_8386763
523	EPI_ISL_9068393
524	EPI_ISL_8450701
525	EPI_ISL_8450706
526	EPI_ISL_9068388
527	EPI_ISL_8450705
528	EPI_ISL_8450700
529	EPI_ISL_8450696
530	EPI_ISL_9068385
531	EPI_ISL_9068381
532	EPI_ISL_8450699

533	EPI_ISL_8450704
534	EPI_ISL_9068386
535	EPI_ISL_8450707
536	EPI_ISL_8450712
537	EPI_ISL_8450711
538	EPI_ISL_9072411
539	EPI_ISL_9068398
540	EPI_ISL_8450709
541	EPI_ISL_8450713
542	EPI_ISL_9068396
543	EPI_ISL_9068397
544	EPI_ISL_9072412
545	EPI_ISL_9068387
546	EPI_ISL_8450695
547	EPI_ISL_8450702
548	EPI_ISL_8450710
549	EPI_ISL_9072410
550	EPI_ISL_8450717
551	EPI_ISL_9068395
552	EPI_ISL_8450716
553	EPI_ISL_9068399
554	EPI_ISL_9068382
555	EPI_ISL_9068383
556	EPI_ISL_9068389
557	EPI_ISL_8450715
558	EPI_ISL_9068384
559	EPI_ISL_9068394
560	EPI_ISL_8450708
561	EPI_ISL_9068390
562	EPI_ISL_9068392
563	EPI_ISL_8450697
564	EPI_ISL_8450703
565	EPI_ISL_8450714
566	EPI_ISL_9068391
567	EPI_ISL_9068400
568	EPI_ISL_8450698
569	EPI_ISL_8450694
570	EPI_ISL_9172089
571	EPI_ISL_9172090
572	EPI_ISL_9172091
573	EPI_ISL_9172092
574	EPI_ISL_9172093
575	EPI_ISL_9172094
576	EPI_ISL_9172095
577	EPI_ISL_9172096

578	EPI_ISL_9172097
579	EPI_ISL_9172098
580	EPI_ISL_9172099
581	EPI_ISL_9172100
582	EPI_ISL_9172101
583	EPI_ISL_9172102
584	EPI_ISL_9172103
585	EPI_ISL_9172104
586	EPI_ISL_9176309
587	EPI_ISL_9172105
588	EPI_ISL_9172106
589	EPI_ISL_9172107
590	EPI_ISL_9172108
591	EPI_ISL_9172109
592	EPI_ISL_9172110
593	EPI_ISL_9172111
594	EPI_ISL_11112486
595	EPI_ISL_11112487
596	EPI_ISL_11112488
597	EPI_ISL_11112489
598	EPI_ISL_11112490
599	EPI_ISL_11112491
600	EPI_ISL_11112492
601	EPI_ISL_11112493
602	EPI_ISL_11112494
603	EPI_ISL_11112495
604	EPI_ISL_11112496
605	EPI_ISL_11112497
606	EPI_ISL_11112498
607	EPI_ISL_11112499
608	EPI_ISL_11112500
609	EPI_ISL_11112501
610	EPI_ISL_11112502
611	EPI_ISL_11112503
612	EPI_ISL_11112504
613	EPI_ISL_11112505
614	EPI_ISL_11112506
615	EPI_ISL_11112507
616	EPI_ISL_11112508
617	EPI_ISL_11112509
618	EPI_ISL_9228150
619	EPI_ISL_9228151
620	EPI_ISL_9228149
621	EPI_ISL_9228155
622	EPI_ISL_9228148

623	EPI_ISL_9228146
624	EPI_ISL_9228162
625	EPI_ISL_9228156
626	EPI_ISL_9228166
627	EPI_ISL_9228161
628	EPI_ISL_9228165
629	EPI_ISL_9228159
630	EPI_ISL_9228167
631	EPI_ISL_9228168
632	EPI_ISL_9228157
633	EPI_ISL_9228163
634	EPI_ISL_9228178
635	EPI_ISL_9228173
636	EPI_ISL_9228176
637	EPI_ISL_9228175
638	EPI_ISL_9228183
639	EPI_ISL_9228169
640	EPI_ISL_9228172
641	EPI_ISL_9228179
642	EPI_ISL_9228182
643	EPI_ISL_9228153
644	EPI_ISL_9228154
645	EPI_ISL_9228158
646	EPI_ISL_9228142
647	EPI_ISL_9228164
648	EPI_ISL_9228185
649	EPI_ISL_9228188
650	EPI_ISL_9228181
651	EPI_ISL_9228160
652	EPI_ISL_9228143
653	EPI_ISL_9228147
654	EPI_ISL_9228180
655	EPI_ISL_9228189
656	EPI_ISL_9228187
657	EPI_ISL_9228152
658	EPI_ISL_9228184
659	EPI_ISL_9228177
660	EPI_ISL_9228145
661	EPI_ISL_9228144
662	EPI_ISL_9228174
663	EPI_ISL_9228171
664	EPI_ISL_9228170

Supplementary Table S1B: Accession numbers of the SARS-CoV-2 sequences with 32≤ C_T ≤39 (N=32).

Sample number	GISAID Accession ID
1	EPI_ISL_5538970
2	EPI_ISL_5538822
3	EPI_ISL_5538942
4	EPI_ISL_5538830
5	EPI_ISL_5538834
6	EPI_ISL_5538950
7	EPI_ISL_5538954
8	EPI_ISL_5538817
9	N/A
10	EPI_ISL_5639444
11	EPI_ISL_5538839
12	N/A
13	EPI_ISL_5538845
14	EPI_ISL_5538851
15	EPI_ISL_5538857
16	EPI_ISL_5639443
17	EPI_ISL_5538867
18	EPI_ISL_5538871
19	EPI_ISL_5538877
20	EPI_ISL_5538883
21	EPI_ISL_5538889
22	EPI_ISL_5538896
23	EPI_ISL_5538965
24	EPI_ISL_5538974
25	N/A
26	EPI_ISL_5538902
27	EPI_ISL_5538907
28	EPI_ISL_5538914
29	EPI_ISL_5538919
30	EPI_ISL_5538924
31	EPI_ISL_5538934
32	EPI_ISL_5538962

With N/A: not applicable.

Supplementary Table S2: SARS-CoV-2 strains included in the phylogenetic tree.

Clade	VOC	Number of sequences
19A	non-VOC	Original Wuhan Virus: GenBank reference NC045512.2
20D	non-VOC; C36.3.1	Study sequence (N=1)<br>GenBank reference OU351896
20I	Alpha (V1); B.1.1.7	Study sequences (N=206)<br>GenBank reference OU011713
20H	Beta (V2); B.1.351	Study sequences (N=2)<br>GenBank reference OM739433
20J	Gamma (V3); P.1	GenBank reference MZ020420
21I	Delta; B.1.617.2	Study sequences (N=269)*<br>GenBank reference OV342852
21J	Delta; B.1.617.2	Study sequences (N=56)**<br>GenBank reference OU595087
21K	Omicron, BA.1	Study sequences (N=124)<br>GenBank reference ON157743
21L	Omicron, BA.2	Study sequences (N=2)<br>GenBank reference ON150103

With * sublineages: AY.43 (N=106), AY.4 (N=47), AY.122 (N=37), B.1.617.2 (N=15), AY.5 (N=12), AY.129 (N=10), AY.126 (N=8), AY.42 (N=6), AY.46 (N=6), AY.121 (N=5), AY.98 (N=3), AY.125 (N=3), AY.33 (N=2), AY.123 (N=2), AY.124 (N=2), AY.34 (N=1), AY.36 (N=1), AY.109 (N=1), AY.111 (N=1), AY.118 (N=1), and ** sublineages: AY.9 (N=46), AY.70 (N=9), AY.71 (N=1).

Supplementary Table S3: Validation of RT-PCR genotyping assays of the TaqMan SARS-CoV-2 mutation panel using a panel of reference samples (N=13) provided by the National Institute for Public Health and the Environment (RIVM) containing SARS-CoV-2 positive samples with known whole genome sequences.

**Reference SARS-CoV-2 samples provided by the RIVM**					**Spike protein mutations**										**Conclusion**	
**Sample**	**Viral load (TCID50/mL)**	***E* gene(1) (C_T value)**	***RdRp* gene(1) (C_T value)**	**Pango lineage (WGS)**	**N501Y**	**E484K**	**K417N**	**K417T**	**del69_70**	**P681H**	**delY144**	**A701V**	**L452R**	**Q493R**	**WHO label WGS**	**WHO label RT-PCR**
**20159/2021**	**1.78E+07**	**17.29**	**17.56**	**B.1.351**	**mut**	**mut**	**mut**	*****	**wt**	**wt**	**wt**	**mut**	**wt**	**x**	**Beta**	**Beta**
**10915/2021**	**7.50E+04**	**18.84**	**18.97**	**P.1**	**mut**	**mut**	*****	**mut**	**wt**	**wt**	**wt**	**wt**	**wt**	**x**	**Gamma**	**Gamma**
**10706/2021**	**1.78E+05**	**17.89**	**17.62**	**P.2**	**wt**	**mut**	**wt**	**wt**	**wt**	**wt**	**wt**	**wt**	**wt**	**x**	**Zeta**	**Zeta**
**11401/2021**	**5.62E+04**	**17.97**	**17.57**	**B.1.429**	**wt**	**wt**	**wt**	**wt**	**wt**	**wt**	**wt**	**wt**	**mut**	**x**	**Epsilon**	**Epsilon**
**20274/2020**	**2.37E+06**	**18.27**	**18.14**	**B.1.258.21**	**wt**	**wt**	**wt**	**wt**	**mut**	**wt**	**wt**	**wt**	**wt**	**x**	**-**	**-**
**20637/2020**	**5.62E+05**	**19.33**	**20.00**	**B.1.1.7**	**mut**	**wt**	**wt**	**wt**	**mut**	**mut**	**mut**	**wt**	**wt**	**x**	**Alpha**	**Alpha**
**20300/2020**	**1.33E+06**	**18.81**	**18.51**	**B.1.177**	**wt**	**wt**	**wt**	**wt**	**wt**	**wt**	**wt**	**wt**	**wt**	**x**	**-**	**-**
**12844/2021**	**3.16 E+05**	**18.82**	**19.47**	**B.1.1.7**	**mut**	**mut**	**wt**	**wt**	**mut**	**mut**	**mut**	**wt**	**wt**	**wt**	**Alpha**	**Alpha**
**13806/2021**	**1.00 E+06**	**18.28**	**18.67**	**B.1.526**	**wt**	**mut**	**wt**	**wt**	**wt**	**wt**	**wt**	**mut**	**wt**	**wt**	**Iota**	**Iota**
**15598/2021**	**7.50 E+05**	**19.57**	**19.72**	**B.1.525**	**wt**	**mut**	**wt**	**wt**	**mut**		**mut**	**wt**	**wt**	**wt**	**Eta**	**Eta**
**27142/2021**	**2.37 E+05**	**17.46**	**18.82**	**B.1.617.2**	**wt**	**wt**	**wt**	**wt**	**wt**	*****	**wt**	**wt**	**mut**	**wt**	**Delta**	**Delta**
**71076/2021**	**3.38 E+03**	**24.40**	**?**	**B.1.1.529**	**mut**	*****	**mut**	*****	**mut**	**mut**	**/**	**mut**	**wt**	**mut**	**Omicron**	**Omicron**
**72291/2021**	**6.00 E+02**	**23.60**	**?**	**B.1.1.529**	**mut**	*****	**mut**	*****	**mut**	**mut**	**/**	**wt**	**wt**	**mut**	**Omicron**	**Omicron**

With mut: mutation, wt: wildtype, x: not tested, -: no WHO label assigned, ?: not provided by the RIVM, *: the presence of another mutation at the same position would cause the assay to show a result which can look as a heterozygous call and can therefore signal the presence of a different mutation at that position, /: the delY144 assay was designed to detect the corresponding mutation in the Alpha variant of concern and could not be applied for the Omicron variant due to additional mutations being present in the adjacent positions that would compromise the ability of probes and primers to bind appropriately.

References

1. Corman VM, Landt O, Kaiser M, Molenkamp R, Meijer A, Chu DKW, Bleicker T, Brünink S, Schneider J, Schmidt ML, Mulders DGJC, Haagmans BL, Van Der Veer B, Van Den Brink S, Wijsman L, Goderski G, Romette JL, Ellis J, Zambon M, Peiris M, Goossens H, Reusken C, Koopmans MPG, Drosten C. 2020. Detection of 2019 novel coronavirus (2019-nCoV) by real-time RT-PCR. Eurosurveillance 25.
